# Comparative Efficacy and Safety of Echocardiography Versus Radiation Guidance in Percutaneous Balloon Mitral Valvuloplasty: A Retrospective Study

**DOI:** 10.31083/RCM46811

**Published:** 2025-09-22

**Authors:** Ying’ao Zhao, Yiming Yan, Wenchao Li, Ziping Li, Hang Li, Jianing Cui, Junke Chang, Fengwen Zhang, Fang Fang, Qi Li, Wenbin Ouyang, Xiangbin Pan

**Affiliations:** ^1^Department of Structural Heart Disease, National Center for Cardiovascular Disease, China & Fuwai Hospital, Chinese Academy of Medical Sciences & Peking Union Medical College, 100037 Beijing, China; ^2^Pediatric Cardiac Surgery, Fuwai Central China Cardiovascular Hospital, Henan Provincial People’s Hospital, Zhengzhou University People’s Hospital, 450000 Zhengzhou, Henan, China

**Keywords:** mitral stenosis (MS), percutaneous balloon mitral valvuloplasty (PBMV), echocardiography-only-guided

## Abstract

**Background:**

Percutaneous balloon mitral valvuloplasty (PBMV) is the preferred treatment for selected patients with rheumatic mitral stenosis (MS). Although prior research has established the feasibility and safety of echocardiography-guided PBMV, this study aimed to compare the mid- to long-term clinical outcomes and safety profiles between echocardiography-guided and conventional fluoroscopy-guided approaches.

**Methods:**

Consecutive patients who underwent successful PBMV from January 2016 to December 2022 were enrolled. Participants were stratified into two groups based on procedural guidance method: echocardiography-guided and conventional fluoroscopy-guided. The primary outcome of this study was the success of PBMV, and the secondary outcome was a composite of all-cause mortality, reoperation for mitral valve surgery, or repeat PBMV after discharge. Statistical analyses included the Kaplan–Meier survival analysis with log-rank tests and propensity score matching to adjust for confounding factors.

**Results:**

A total of 429 patients underwent PBMV, with 71 (16.6%) in the echo-guided group and 358 (83.4%) in the conventional fluoroscopy-guided group. A success rate of 98.6% was demonstrated in the echocardiography-guided group, and 98.9% in the fluoroscopy-guided group after propensity score match (*p *= 0.84). During follow-up, nine (14.3%) patients in the echo-guided group required surgical intervention, and 13 (10.4%) in the fluoroscopy-guided group; one (1.6%) patient in the echocardiography-guided group and six (4.8%) in the fluoroscopy-guided group died. No significant differences were observed in freedom from re-intervention (*p *= 0.33) and survival (*p *= 0.23).

**Conclusions:**

For selected patients with rheumatic MS, echocardiography-guided PBMV demonstrated an equivalent mid- to long-term efficacy and safety profile compared to fluoroscopy-guided approaches.

## 1. Introduction

Mitral stenosis (MS) is a prevalent outcome of rheumatic heart disease (RHD), which remains a significant health challenge, particularly in developing countries [1]. This condition affects a large portion of the population, causing MS and consequently reducing quality of life while increasing the risk of severe cardiovascular complications, including heart failure, stroke, and arrhythmia [2, 3]. Percutaneous balloon mitral valvuloplasty (PBMV) stands out as an effective treatment for MS, offering a less invasive approach with shorter procedure times and greater cost-effectiveness compared to surgical commissurotomy [4]. First introduced in 1984, PBMV gained widespread adoption after four decades of practice, achieving procedural success rates exceeding 90% and consistently increasing the mitral valve area (MVA) to more than 2.0 cm^2^ [5, 6]. However, the radiation-dependent guiding approach poses extra risks for vulnerable populations, such as pregnant patients, individuals with iodinated contrast allergies, or those with chronic kidney disease, thereby limiting procedural safety and accessibility.

Echocardiography is a crucial part of cardiac interventional procedures, utilized for both intraoperative adjustments and postoperative assessments. Our prior studies have established the safety and efficacy of performing various cardiac interventions solely under echocardiographic guidance [7]. Thus, to extend and ensure the safety of the PBMV procedure, we have endeavored to conduct PBMV using echocardiographic guidance exclusively, thereby mitigating the risks of radiation exposure for both medical staff and patients. Despite this, limited evidence exists on the safety and efficacy of PBMV when conducted under echocardiographic guidance alone. This study aimed to evaluate the mid- to long-term effectiveness and safety of PBMV using different guidance methods, providing evidence-based recommendations for optimizing patient selection and procedural safety.

## 2. Methods

### 2.1 Study Design and Population

This retrospective study evaluated the mid- to long-term efficacy and safety of PBMV using various guidance methods. The study cohort comprised 429 consecutive symptomatic patients with moderate to severe MS (MVA <1.5 cm^2^) who underwent a PBMV procedure between January 2016 and December 2022.

Exclusion criteria in this study encompassed patients with severe organic heart disease, including but not limited to congenital heart disease, coronary atherosclerotic heart disease, other severe valvular diseases, and atrioventricular block, alongside those with malignant tumors or severe systemic diseases, and cases with missing clinical data or those lost to follow-up.

Participants were divided into two groups: echocardiography-guided and fluoroscopy-guided, based on the method of guidance used. The study protocol was approved by the ethics committee of Fuwai Hospital, Chinese Academy of Medical Sciences (Approval No. 2023-2221). Individual informed consent was waived for this retrospective study, which utilized anonymized data.

### 2.2 Procedures

**Fig. 1.**
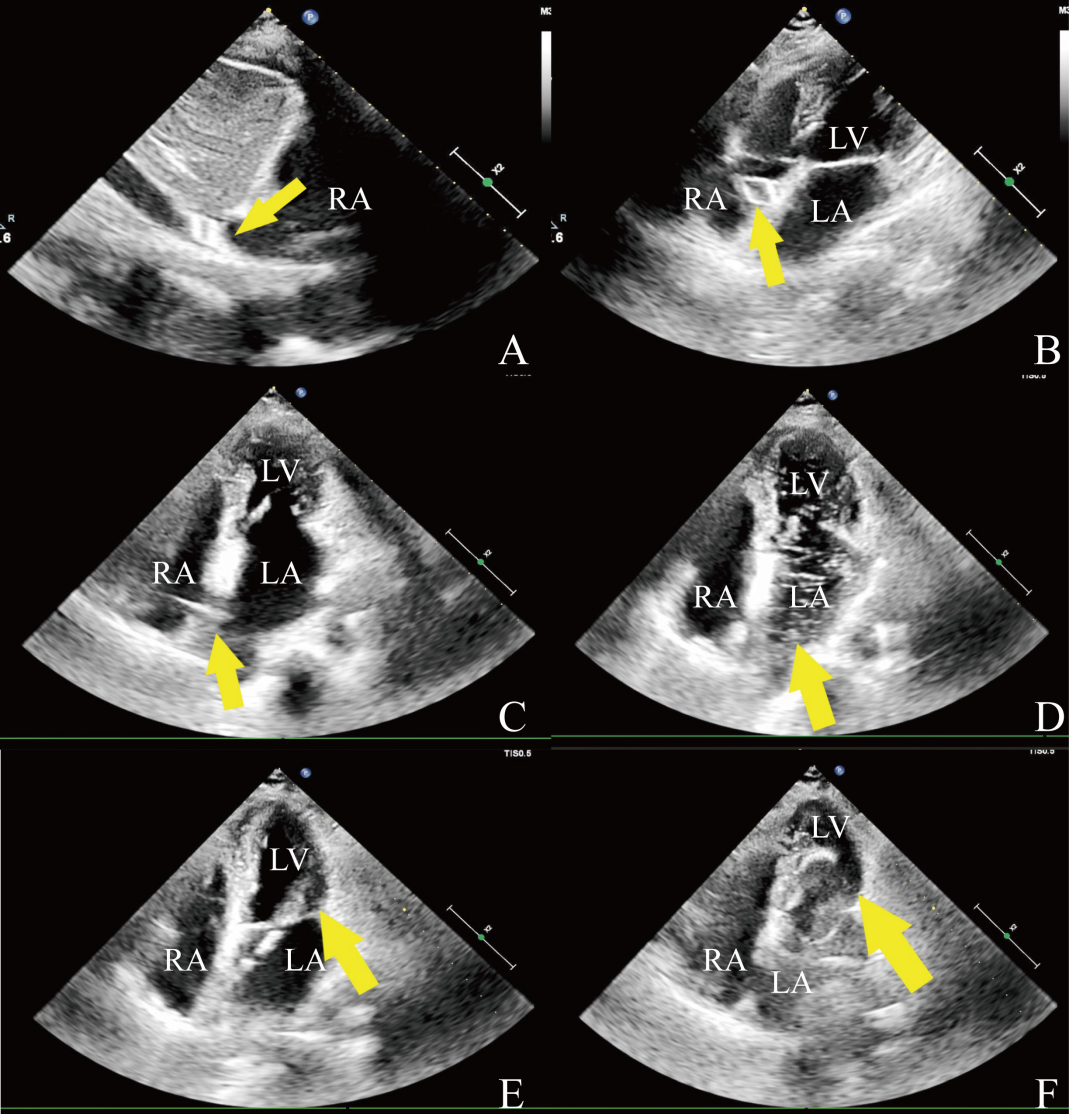
**Intraprocedural echocardiography**. (A) The wire of the spindle-shaped tip (arrow) in the inferior vena cava. (B) The wire (arrow) is in the right atrium. (C) Tent-like deformation (arrow). (D) Injected saline to confirm access of the catheter tip (arrow) into the left atrium. (E) The catheter passed through (arrow) the stenotic mitral valve. (F) The valvuloplasty balloon (arrow) is inflated across the mitral valve. RA, right atrium; LA, left atrium; LV, left ventricle.

Echocardiography-guided PBMV was conducted by physicians in either general or hybrid operating rooms. Transthoracic echocardiography (TTE) was utilized to reassess the status of MS and to provide imaging guidance throughout the procedure. When TTE imaging was suboptimal, transesophageal echocardiography (TEE) was implemented, noting that TEE necessitates sedation or general anesthesia. The procedure commenced with local anesthesia, followed by puncture of the right femoral vein. Guided by echocardiography, a transseptal puncture sheath and needle were steered along a guidewire. The puncture site was determined by the characteristic tent-like deformation of the interatrial septum visible on echocardiography (Fig. 1C). To verify proper access to the atrial septum, echocardiographic visualization of opacified saline bubbles was performed in the left atrium after the injection of 10 mL of heparinized saline through the sheath (Fig. 1D). An Inoue balloon was then introduced into the left atrium, navigated across the mitral annulus, and into the left ventricle under echocardiographic guidance (Fig. 1E). The balloon was swiftly inflated to dilate the mitral valve once positioned at the valve orifice (Fig. 1F). After achieving full inflation of the balloon waist, the balloon was promptly deflated and carefully retracted back into the left atrium. Echocardiography was employed to assess the effectiveness of the dilation.

For PBMV under traditional radiation guidance, the procedure was performed using the Inoue balloon technique in the catheterization laboratory. After local anesthesia, a puncture was made in the right femoral vein, and the guidewire was introduced through the vein into the superior vena cava. The Mullin sheath was advanced along the guidewire, and the Brockenbrough needle was inserted under fluoroscopic guidance, with the needle tip positioned outside the sheath. Once the puncture site was confirmed, the sheath tip was positioned at the fossa ovalis. Blood was aspirated through the needle, and a contrast agent was injected to verify access to the left atrium. Heparin (50–100 U/kg) was administered, and the activated clotting time was maintained above 250 seconds. A septal dilator was used to expand the puncture site, followed by the insertion of the mitral balloon catheter. After the balloon was placed in the left atrium, the metallic extension tube and guidewire were removed, and left atrial pressure was measured. The balloon was advanced to the mitral valve, inflated, and gently repositioned into the left ventricle to ensure no chordae tendineae were crossed. A contrast agent was injected to inflate the balloon, and once fully inflated, the balloon was withdrawn back into the left atrium [6]. Pre- and post-procedure echocardiographic examinations were performed for assessment.

### 2.3 Outcomes and Follow-up

The primary outcome of this study was the success of PBMV, defined as a post-procedural MVA of ≥1.5 cm^2^ or a ≥50% increase in MVA, with mitral regurgitation (MR) less than grade 2+ and no occurrences of death, stroke, mitral surgery, or cardiac tamponade [8]. The secondary outcome was a composite of all-cause mortality, reoperation for mitral valve surgery, or repeat PBMV after discharge. Consequently, baseline data, as well as pre- and post-operative echocardiographic parameters, including left atrial diameter (LAD), left ventricular end-diastolic diameter (LVEDD), left ventricular ejection fraction (LVEF), MVA, transmitral E peak velocity (Emax), and mean transmitral gradient (MTG), were collected and analyzed (**Supplementary Table 1**).

All patients were followed up in the outpatient department. Echocardiography and electrocardiography were performed and assessed at 1, 6, 12 months following the procedure, and annually thereafter.

### 2.4 Statistical Analysis

Missing data for variables with missing values were imputed using MissForest [9]—a random forest imputation algorithm for mixed-type data implemented in R (R ×64 version 4.3.2, R Foundation for Statistical Computing, Vienna, Austria). The missing rates for the study variables are presented in **Supplementary Table 2**.

Categorical variables are expressed as both numbers and percentages, while continuous variables are depicted using the mean (with standard deviation) or median (with interquartile range), depending on the data distribution. For the comparison of categorical variables, the chi-square test or Fisher’s exact test was employed. Meanwhile, the *t*-test or the Mann–Whitney U test was utilized for continuous variables.

To mitigate potential baseline confounders, a propensity score matching (PSM) analysis was conducted. Balanced variables for matching included gender, age, body mass index (BMI), atrial fibrillation, New York Heart Association classification (NYHA), LAD, LVEDD, LVEF, MVA, Emax, MTG, tricuspid regurgitation (TR), Wilkins score, pregnancy, and chronic kidney diseases (CKDs) as covariates, 0.01 as the caliper width, and a 1:2 match was chosen to adjust cases of the two groups to reduce the bias. The absolute standardized difference (ASD) was used for between-group comparisons, with an ASD <10% considered ideal [10]. The absolute standardized difference for pre-PSM and post-PSM is presented in **Supplementary Fig. 1**.

The Kaplan–Meier analysis was used to determine survival and event-free rates, and the log-rank test was performed to compare differences between the two groups. A *p*-value < 0.05 was set to denote statistical significance. The statistical analyses were performed using SPSS version 26.0 (IBM Corporation, Armonk, NY, USA).

## 3. Results

A total of 429 patients who underwent PBMV from January 2016 to December 2022 were included in this study, comprising 71 patients in the echocardiography-guided group and 358 patients in the fluoroscopy-guided group. All 429 patients survived the interventions, with an overall PBMV success rate of 98.8%. Each group had one patient who required surgical intervention due to valvuloplasty failure; the success rate was 98.6% in the echocardiography-guided group and 98.9% in the fluoroscopy-guided group (*p* = 0.84).

### 3.1 Baseline Characteristics

Overall, the majority of patients were female (80.4%), with a mean age of 49.3 years and a mean BMI of 23.9 kg/m^2^. Among the cohort, 105 patients (24.5%) had atrial fibrillation, and 26 (6.1%) had severe TR. The mean mitral valve area was 0.95 cm^2^, and the median Wilkins score was 7.0 (5.0, 7.0). Of the 71 patients (16.6%) who underwent PBMV under echocardiographic guidance, 11 (15.5%) were guided by TEE. Clinical characteristics pre- and post-PSM are summarized in Table 1, and the ASD of the between-group covariates after matching was <10%. There were no significant differences in gender, age, BMI, atrial fibrillation, LAD, LVEDD, LVEF, MVA, Emax, MTG, preoperative severe TR, Wilkins score, balloon size, procedure time, hospital stay, or inpatient costs between the two groups. Additionally, a difference in the NYHA classification component was observed between the two groups (*p* = 0.005), with patients undergoing echo-guided PBMV representing a higher percentage in NYHA classes III and IV than the fluoroscopy-guided group before and after PSM. Three (4.92%) pregnant patients and two (3.28%) patients with renal insufficiency successfully underwent echo-guided PBMV.

**Table 1.** **Baseline characteristics of study patients in two groups, pre- vs. post-PSM**.

Characteristics		Pre-PSM			Post-PSM		
		Echo (n = 71)	Ra (n = 358)	*p*-value	Echo (n = 64)	Ra (n = 126)	*p*-value
Female, n (%)		61 (85.9)	284 (79.3)	0.20	54 (84.4)	106 (84.1)	0.96
Age, years		48.6 ± 12.4	49.4 ± 12.6	0.65	48.8 ± 11.9	48.5 ± 12.6	0.85
BMI, kg/m^2^		23.3 ± 3.07	24.0 ± 3.68	0.12	23.4 ± 3.17	23.4 ± 3.21	0.93
AF, n (%)		21 (29.6)	84 (23.5)	0.27	19 (29.7)	37 (29.4)	0.96
NYHA class, n (%)				0.005			0.006
	I	14 (19.7)	35 (9.8)		13 (20.3)	14 (11.1)	
	II	32 (45.1)	236 (65.9)		31 (48.4)	90 (71.4)	
	III	24 (33.8)	84 (23.5)		20 (31.3)	20 (15.9)	
	IV	1 (1.4)	3 (0.8)		0	2 (1.6)	
LAD, mm		46.7 ± 6.04	47.3 ± 6.95	0.45	47.2 ± 5.95	46.8 ± 7.53	0.75
LVEDD, mm		44.9 ± 4.49	44.7 ± 4.56	0.66	45.0 ± 4.31	44.5 ± 4.46	0.47
EF, %		63.8 ± 5.29	63.4 ± 4.83	0.60	63.4 ± 5.35	63.2 ± 4.93	0.79
MVA, cm^2^		0.90 ± 0.21	0.95 ± 0.23	0.065	0.91 ± 0.22	0.89 ± 0.21	0.68
Emax, m/s		2.30 ± 0.45	2.24 ± 0.42	0.23	2.28 ± 0.44	2.26 ± 0.47	0.80
MTG, mmHg		12.6 ± 6.02	11.5 ± 5.6	0.10	12.4 ± 5.89	12.3 ± 5.60	0.93
Severe TR, n (%)		5 (7.0)	21 (5.9)	0.78	5 (7.8)	9 (7.1)	1.00
Wilkins score		6.0 [5.0, 7.0]	7.0 [6.0, 8.0]	0.15	6.5 [5.0, 7.0]	6.0 [5.0, 8.0]	0.62
Pregnancy, n (%)		3 (4.23)	0	0.004	0	0	NA
CKD, n (%)		2 (2.82)	0	0.027	0	0	NA
Ballon size, mm		26.0 [26.0, 27.0]	26.0 [26.0, 26.0]	0.83	26.0 [26.0, 27.0]	26.0 [26.0, 26.0]	0.18
Procedure time, min		63.7 ± 12.7	64.0 ± 33.5	0.94	63.2 ± 12.3	63.2 ± 27.3	0.99
Hospital stay, days		4.0 [3.0, 5.0]	4.0 [3.0, 5.0]	0.26	4.0 [3.0, 5.0]	4.0 [3.0, 5.0]	0.31
Inpatient cost, RMB		43,172.0 ± 3956.2	43,268.8 ± 6860.6	0.91	43,178.0 ± 3902.6	43,431.8 ± 6698.3	0.78

Data are presented as the mean ± SD, n (%), or median [IQR]. PSM, propensity score matching; Echo, echocardiography-guided; Ra, radiation-guided; BMI, body mass index; AF, atrial fibrillation; NYHA, New York Heart Association; LAD, left atrial diameter; LVEDD, left ventricular end-diastolic diameter; LVEF, left ventricular ejection fraction; MVA, mitral valve area; Emax, transmitral E peak velocity; MTG, mean transmitral gradient; TR, tricuspid regurgitation; CKD, chronic kidney disease; NA, not applicable. The exchange rate of CNY to USD is ¥7.1029 = $1.

### 3.2 Procedural Outcomes

**Fig. 2.**
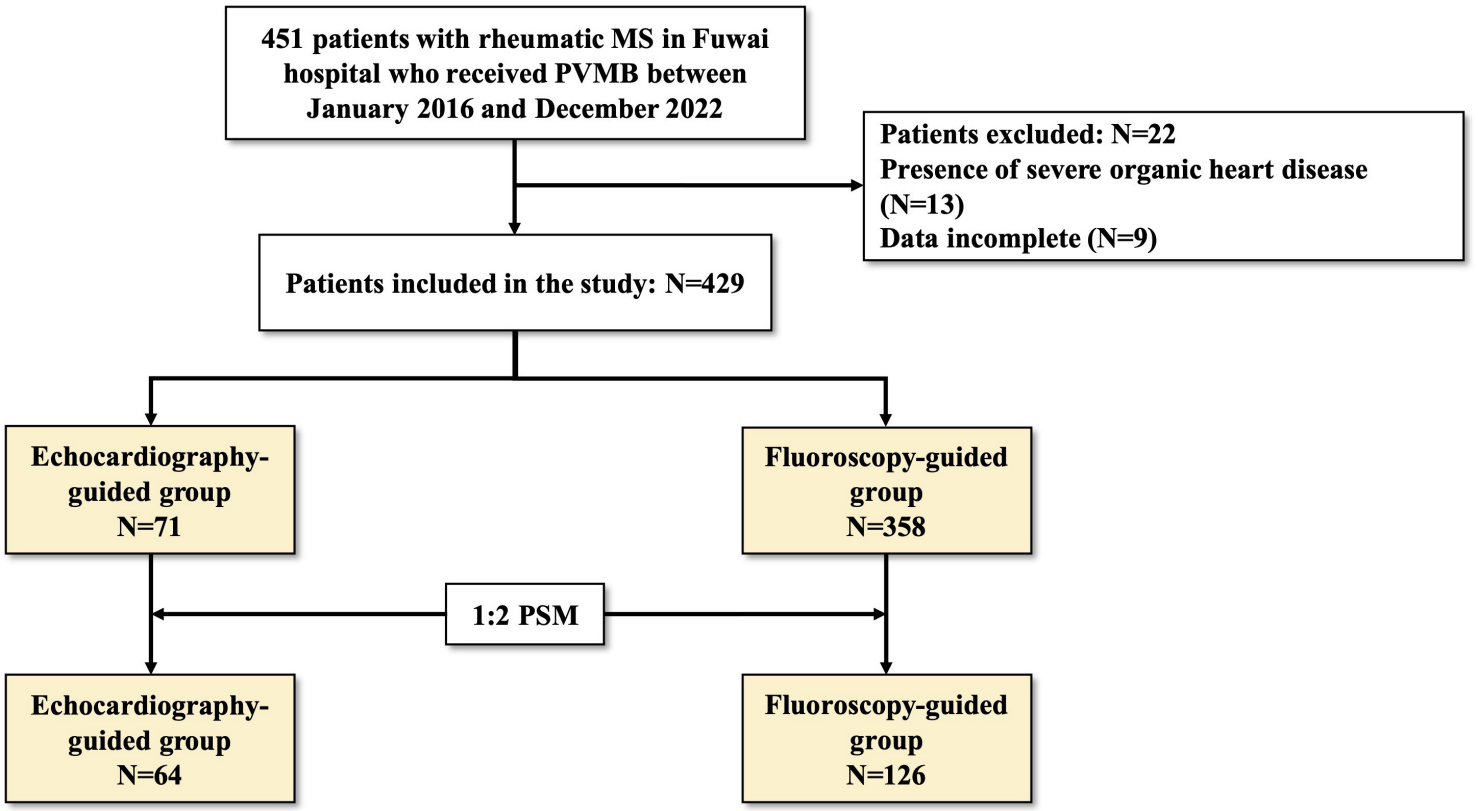
**Flow of study population**. MS, mitral stenosis; PBMV, percutaneous balloon mitral valvuloplasty; MR, mitral regurgitation; PSM, propensity score matching.

After propensity score matching, the echocardiography-guided group had one patient (1.6%) who required surgical intervention, while the fluoroscopy-guided group had one patient (0.8%) with severe mitral regurgitation (MR) intra-operatively. Postoperative echocardiography data are summarized in Table 2. MVA was significantly larger in the echocardiography-guided group compared with the fluoroscopy-guided group (1.68 ± 0.27 vs*.* 1.56 ± 0.28 cm^2^; *p* = 0.005) (Fig. 2), and the Emax was improved more in the echocardiography-guided group (1.72 ± 0.34 vs*.* 1.84 ± 0.34 m/s; *p* = 0.014). Additionally, MTG was significantly decreased in the echocardiography-guided group (5.04 ± 2.21 vs*.* 6.13 ± 2.43 m/s; *p* = 0.003).

**Table 2.** **Postoperative outcomes of study patients**.

Outcomes	Pre-PSM		*p*-value	Post-PSM		*p*-value
	Echo (n = 71)	Ra (n = 358)		Echo (n = 64)	Ra (n = 126)	
LAD, mm	45.1 (7.21)	45.3 (6.95)	0.81	45.3 (7.54)	45.4 (8.08)	0.93
LVEDD, mm	45.8 (3.93)	45.5 (4.12)	0.53	45.8 (4.09)	45.3 (4.04)	0.40
EF, %	64.6 (5.20)	64.7 (4.71)	0.94	64.2 (5.10)	64.0 (4.65)	0.84
MVA, cm^2^	1.70 (0.28)	1.60 (0.27)	0.003	1.68 (0.27)	1.56 (0.28)	0.005
Emax, m/s	1.70 (0.33)	1.81 (0.32)	0.012	1.72 (0.34)	1.84 (0.34)	0.014
MTG, mmHg	5.18 (2.19)	6.09 (2.38)	0.003	5.04 (2.21)	6.13 (2.43)	0.003
Severe TR	5 (7.0)	8 (2.2)	0.047	5 (7.8)	6 (4.8)	0.51

Data are presented as the mean (SD) or n (%). PSM, propensity score matching; Echo, echocardiography-guided; Ra, radiation-guided; LAD, left atrial diameter; LVEDD, left ventricular end-diastolic diameter; LVEF, left ventricular ejection fraction; MVA, mitral valve area; Emax, transmitral E peak velocity; MTG, mean transmitral gradient; TR, tricuspid regurgitation.

### 3.3 Follow-up Outcomes

**Fig. 3.**
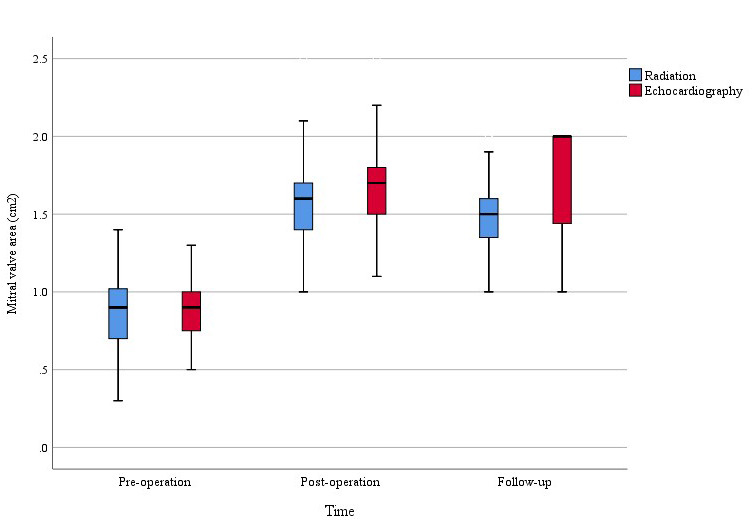
**Preoperative, postoperative, and follow-up changes in MVA of the two groups**. MVA improved significantly in both groups, with more pronounced enhancement noted in the echocardiography-guided group.

**Fig. 4.**
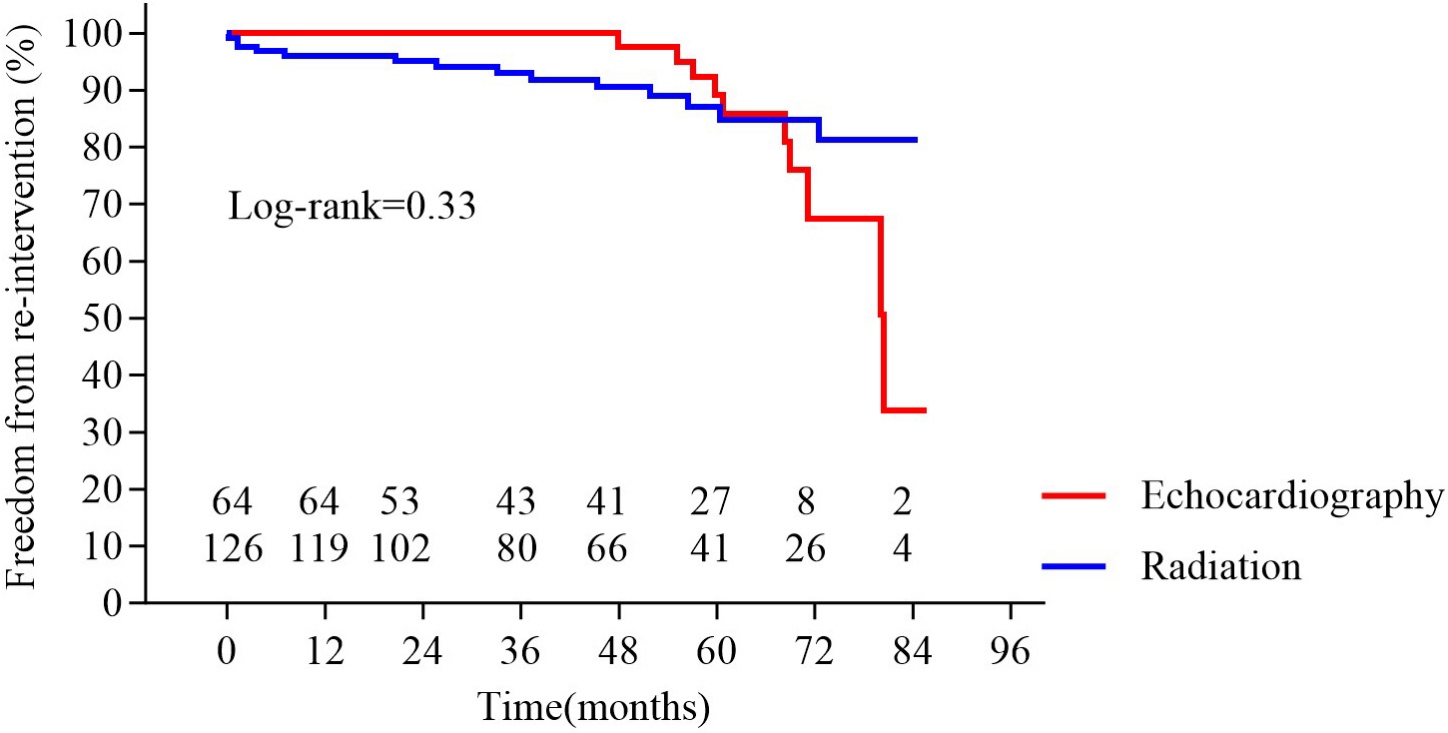
**Freedom from re-intervention in the two groups**. No significant difference was observed between groups (*p* = 0.33).

**Fig. 5.**
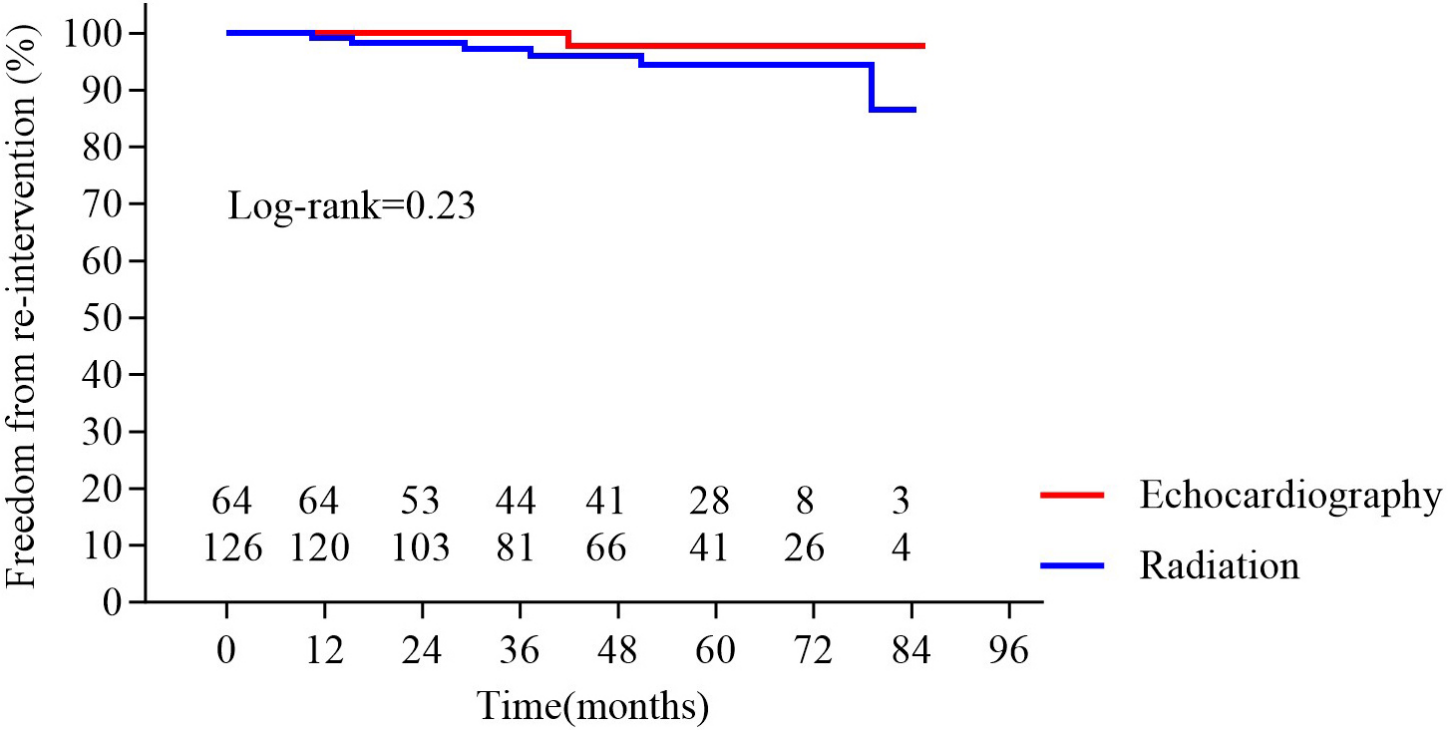
**Survival in the two groups**. No significant difference was observed between groups (*p* = 0.23).

The median follow-up period was 50.6 months, ranging from 3.6 to 84.5 months. During the follow-up, surgery was performed on nine patients (14.3%) in the echocardiography-guided group and 13 patients (10.4%) in the traditional group due to re-stenosis. One patient (1.6%) in the echocardiography-guided group died from heart failure, while six patients (4.8%) in the traditional group died, including two (1.6%) from stroke and four (3.2%) due to heart failure. The related data from these patients were excluded during analysis. Follow-up echocardiographic data are summarized in Table 3. There was a larger MVA in the echocardiography-guided group compared with the fluoroscopy-guided group (1.73 ± 0.41 vs*.* 1.49 ± 0.27 cm^2^; *p* < 0.001; Fig. 3). Kaplan–Meier survival curve analysis showed no significant differences in re-intervention rates (Fig. 4) or survival between the groups at 7 years (Fig. 5).

**Table 3.** **Follow-up outcomes of study patients**.

Outcomes	Pre-PSM		*p*-value	Post-PSM		*p*-value
	Echo (n = 71)	Ra (n = 358)		Echo (n = 54)	Ra (n = 126)	
LAD, mm	43.9 (6.89)	44.9 (6.97)	0.29	44.3 (7.04)	45.0 (7.23)	0.49
LVEDD, mm	45.0 (4.82)	45.6 (3.34)	0.19	45.0 (4.80)	45.5 (3.50)	0.39
EF, %	63.4 (4.67)	63.7 (5.01)	0.71	63.4 (4.62)	63.0 (5.00)	0.59
MVA, cm^2^	1.70 (0.43)	1.50 (0.27)	<0.001	1.73 (0.41)	1.49 (0.27)	<0.001
Emax, m/s	1.89 (0.28)	1.86 (0.26)	0.35	1.87 (0.26)	1.85 (0.17)	0.68
MTG, mmHg	6.20 (2.31)	6.39 (2.55)	0.55	6.10 (2.03)	6.62 (2.68)	0.17
Severe TR	1 (1.4)	4 (1.1)	1.00	1 (1.6)	1 (0.8)	1.00

Data are presented as the mean (SD) or n (%). PSM, propensity score matching; Echo, echocardiography-guided; Ra, radiation-guided; LAD, left atrial diameter; LVEDD, left ventricular end-diastolic diameter; LVEF, left ventricular ejection fraction; MVA, mitral valve area; Emax, transmitral E peak velocity; MTG, mean transmitral gradient; TR, tricuspid regurgitation.

## 4. Discussion

Echocardiography has emerged as an indispensable imaging modality in cardiac interventional procedures, offering distinct advantages in real-time visualization of three-dimensional cardiac anatomy while minimizing the requirement for specialized catheterization laboratory equipment. These inherent benefits establish echocardiography-guided interventions as a clinically valuable strategy [11]. Building on the well-documented efficacy of echocardiographic guidance and considering the specific requirements of the patient population, we implemented an echocardiography-only-guided approach for PBMV, aiming to optimize its clinical utility. This study found no significant difference in procedural success for the mid- to long-term outcomes between the two groups, providing valuable evidence to support the adoption of this innovative method.

Additionally, this study observed a statistically significant difference in postoperative and follow-up MVA between the two groups (Fig. 3). This phenomenon may be attributed to several key factors: (1) Enhanced balloon positioning: Real-time echocardiographic guidance allows for more precise balloon placement and optimal commissural splitting, minimizing the risk of suboptimal dilation. (2) Avoidance of overstretching: Direct visualization of the valve structure helps prevent excessive balloon inflation, reducing the likelihood of leaflet damage or excessive commissural tearing, which may contribute to mitral regurgitation. (3) Individualized dilation strategy: Echocardiography provides immediate feedback on valve morphology and leaflet mobility, enabling tailored balloon sizing and pressure adjustments to achieve an optimal balance between effective dilation and structural integrity. Although the difference between the two groups was small, this difference could suggest that echocardiography guidance may have a more favorable long-term effect in the interventional treatment of MS, highlighting the advantages of echocardiography in procedural aspects of intervention. However, further studies with longer follow-up periods and larger sample sizes are needed to investigate and validate this conclusion. Furthermore, this study demonstrated that the echocardiography-guided group can effectively manage surgical treatment for patients in more compromised conditions. In the echocardiography-guided group, a higher proportion of patients were classified as NYHA functional classes III or IV, indicating a potential advantage of echocardiographic guidance in managing critically ill patients. Nonetheless, this observation is based on a limited sample size, and further studies with larger cohorts are necessary to confirm these findings.

Since the inception of PBMV in 1991, this treatment has evolved into the gold-standard Class I recommendation for symptomatic rheumatic MS in anatomically suitable patients, as endorsed by the American College of Cardiology/American Heart Association (ACC/AHA) guidelines [12]. Radiation exposure presents significant risks, particularly to fetuses, and should not be underestimated, as it can lead to fetal deformities and tumor development [13]. Protective measures, such as using lead aprons to shield the abdomen and reducing radiation exposure time, are insufficient to fully mitigate the adverse effects of radiation on pregnant women and their fetuses. Furthermore, contrast agents can induce allergies and renal failure [14]. Hence, replacing radiation with echocardiography for interventional procedures provides a safer alternative for patients with these conditions, safeguarding both medical staff and patients. In this study, three pregnant patients and two patients with renal insufficiency who underwent PBMV under echocardiographic guidance showed marked improvements in hemodynamics and clinical symptoms, further validating the applicability of the technology in special patient populations.

Notably, operators primarily rely on specific anatomical landmarks for positioning and execution in traditional interventional procedures. However, echocardiography offers enhanced visualization, significantly improving procedural accuracy. Moreover, echocardiography enables precise identification of the optimal puncture site during interatrial septal puncture. In both the apical four-chamber and parasternal short-axis views, the characteristic “tent-like” deformation of the interatrial septum revealed the puncture needle tip, allowing real-time adjustments. This facilitates optimization of the distance between the puncture site and the mitral valve orifice based on left atrial size, ensuring the smooth passage of the balloon. More importantly, echocardiography provides a clear delineation of the mitral valve annular plane, ensuring precise balloon positioning at the annular level. This accuracy minimizes the risk of balloon misplacement in the left ventricle or left atrium, a potential limitation of fluoroscopic guidance [15, 16]. Moreover, RHD remains highly prevalent in developing nations and low- to middle-income countries [17], where the economic feasibility of ultrasound machines offers a significant advantage over catheterization laboratories. Echocardiography-guided PBMV can enhance accessibility and utilization in these resource-limited settings, potentially improving procedural efficacy and expanding the reach of life-saving interventions.

In this study, one patient in the echocardiography-guided group developed mitral valve rupture due to severe leaflet fibrosis, thickening, and adhesion, resulting in chordal rupture at the P2 segment and subsequent severe MR, which required surgical intervention on the third postoperative day. This case underscores the importance of a thorough preoperative assessment, particularly for patients with severe valvular calcification, to minimize the risk of surgical intervention. Echocardiography, which visualizes structures based on their reflective surfaces, may not delineate the catheter and guidewire tip, presenting a significant learning curve due to the differences between echocardiographic and radiation guidance. Therefore, selecting a guidewire detectable by echocardiography is crucial. Indeed, a guidewire with a spindle-shaped tip is more visible under echocardiography [18], facilitating the deployment of the wire and catheter (Fig. 1A,B).

## 5. Study Limitations

In our study, the absence of systematically collected patient-reported outcomes represents a key study limitation. While echocardiographic and clinical endpoints provide objective measures of efficacy, these endpoints cannot fully capture the impact of the treatment on symptom burden, functional status, or health-related quality of life, particularly in this young patient population. Future studies should integrate validated tools, such as the MLHFQ and KCCQ, administered at standardized intervals, to contribute to an improved comprehensive comparison of the effectiveness of different percutaneous balloon mitral valvuloplasty guidance methods, especially with regard to their impact on patient well-being.

## 6. Conclusions

For patients with rheumatic MS, our study demonstrates that clinical outcomes, including postoperative, mid-term, and long-term mortality and complications, are comparable between PBMV performed under echocardiography guidance and traditional guidance. This approach preserves the minimally invasive and safety profiles of traditional percutaneous interventional treatments while avoiding the risks associated with radiation and contrast agents.

## Availability of Data and Materials

The data sets generated and analyzed during the current study are not publicly available due to regulation of Ethics Committee, but are available from the corresponding authors on reasonable request.

## Supplementary Material

Supplementary material associated with this article can be found, in the online version, at https://doi.org/10.31083/RCM46811.
