# Supplementary material for: Comparative Efficacy and Safety of Echocardiography Versus Radiation Guidance in Percutaneous Balloon Mitral Valvuloplasty: A Retrospective Study
Source: Rev Cardiovasc Med. 2025 Sep 22;26(9):46811. doi: 10.31083/RCM46811 (PMC12516735; doi:10.31083/RCM46811)
Supplement: Supplementary file 1 [file 2153-8174-26-9-46811-s1.docx]

**Supplemental Material**

*Comparative Efficacy and Safety of Echocardiography versus Radiation Guidance in Percutaneous Balloon Mitral Valvuloplasty: A Retrospective Study*

Content of Supplemental Material

Supplemental Tables

- **Supplementary Table 1.** Definition of study variables.
- **Supplementary Table 2**. Missing rates of study variables.
- **Supplementary Fig. 1**. The absolute standardized differences of pre- and post-PSM.

**Supplementary Table 1.** Definition of study variables.

|  | Definition | Continuous/Categorical/Binary |
| --- | --- | --- |
| Age | Years after birth | Continuous |
| Sex | Female and Male | Binary, Female/Male |
| BMI | Calculated by BMI Equation | Continuous, kg/m^2^ |
| AF | Atrial fibrillation | Binary, Yes/No |
| Left atrial diameter | Left atrial diameter measured by transthoracic echocardiography | Continuous, in mm |
| LVEDD | Left ventricle diastolic diameter measured by transthoracic echocardiography | Continuous, in mm |
| MVA | Mitral valve area measured by transthoracic echocardiography | Continuous, in cm^2^ |
| Emax | Transmitral E peak velocity measured by transthoracic echocardiography | Continuous, in m/s |
| MTG | Mean transmitral gradient measured by transthoracic echocardiography | Continuous, in mmHg |
| TR | Tricuspid valve regurgitation measured by transthoracic echocardiography | Binary, Yes/No |
| Pregnancy | Admission pregnant status | Binary, Yes/No |
| CKD | Admission chronic kidney disease history | Binary, Yes/No |

**Abbreviations:** BMI, body mass index; LVEDD, Left ventricle end-diastolic diameter; MVA, Mitral valve area; Emax, Transmitral E peak velocity; MTG, Mean transmitral gradient; TR, Tricuspid valve regurgitation; CKD, chronic kidney disease.

**Supplementary Table 2** Missing rates of study variables.

| Variables | Missing, N (%) |
| --- | --- |
| BMI | 17 (3.96) |
| Left atrial diameter | 14 (3.26) |
| LVEDD | 14 (3.26) |
| EF | 9 (2.10) |
| MVA | 4 (0.93) |
| Emax | 15 (3.50) |
| MTG | 24 (5.59) |
| Ballon size | 4 (0.93) |

**Abbreviations:** BMI, body mass index; LVEDD, left ventricular end-diastolic diameter; EF, left ventricular ejection fraction; MVA, mitral valve area; Emax, transmitral E peak velocity; MTG, mean transmitral gradient.


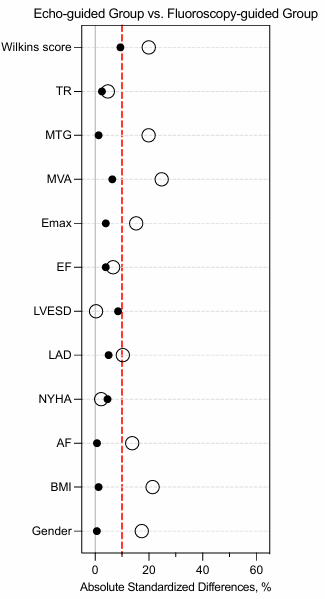

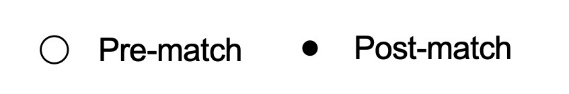


**Supplementary Fig. 1** The standardized difference of pre-PSM. BMI, body mass index; LAD,left atrial diameter; LVEDD, left ventricular end-diastolic diameter; EF, left ventricular ejection fraction; MVA, mitral valve area; Emax, transmitral E peak velocity; MTG, mean transmitral gradient; TR, tricuspid regurgitation.
